# Biochemical Changes Induced by the Administration of *Cannabis sativa* Seeds in Diabetic Wistar Rats

**DOI:** 10.3390/nu15132944

**Published:** 2023-06-28

**Authors:** Camelia Munteanu, Mihaela Mihai, Francisc Dulf, Andreea Ona, Leon Muntean, Floricuța Ranga, Camelia Urdă, Daria Pop, Tania Mihaiescu, Sorin Marian Mârza, Ionel Papuc

**Affiliations:** 1 Department of Plant Culture, Faculty of Agriculture, University of Agricultural Sciences and Veterinary Medicine Cluj-Napoca, Calea Mănăştur 3-5, 400372 Cluj-Napoca, Romania; camelia.munteanu@usamvcluj.ro (C.M.); leon.muntean@usamvcluj.ro (L.M.); 2 Department of Transversal Competencies, University of Agricultural Sciences and Veterinary Medicine Cluj-Napoca, Calea Mănăştur 3-5, 400372 Cluj-Napoca, Romania; mihaela.mihai@usamvcluj.ro; 3 Department of Environmental and Plant Protection, Faculty of Agriculture, University of Agricultural Sciences and Veterinary Medicine Cluj-Napoca, Calea Mănăştur 3-5, 400372 Cluj-Napoca, Romania; francisc.dulf@usamvcluj.ro (F.D.); tania.mihaiescu@usamvcluj.ro (T.M.); 4 Department of Food Science, University of Agricultural Sciences and Veterinary Medicine Cluj-Napoca, Calea Mănăştur 3-5, 400372 Cluj-Napoca, Romania; floricutza_ro@yahoo.com; 5 Agricultural Research Development Station Turda, 27 Agriculturii Street, 401100 Turda, Romania; camelia.urda@scdaturda.ro; 6 Clinic of Obstetrics and Gynecology II “Dominic Stanca”, University of Medicine and Pharmacy “Iuliu Hațieganu” Cluj-Napoca, Victor Babeș 8, 400347 Cluj-Napoca, Romania; drdariamariapop@gmail.com; 7 Faculty of Veterinary Medicine, University of Agricultural Sciences and Veterinary Medicine Cluj-Napoca, Calea Mănăştur 3-5, 400372 Cluj-Napoca, Romania; ionel.papuc@usamvcluj.ro

**Keywords:** cannabis, hydroxybenzoic acid, phenolic activity, fatty acids, glycemia

## Abstract

The present pilot study investigates the blood biochemical changes induced by hemp seeds in rats with diabetes. The composition of industrial hemp seeds, antioxidant activity, identification and quantification of phenols and fatty acids from hemp oil were determined. The Wistar adult rats used in the experiment were divided into three groups (*n* = 6) and kept under standard conditions. Group one, the control group (individuals without diabetes), and group two (diabetic individuals) received water and normal food ad libitum, while the third group, also including diabetic individuals, received specific food (hemp seeds) and water ad libitum. Subsequent blood biochemical parameters were determined. Hemp seeds had higher phenol (14 compounds), flavonoids and PUFA contents compared to other plants seeds. In addition, the antioxidant activity in *Cannabis sativa* was also increased. Moreover, the ratio between *n*-6 and *n*-3 was 4.41, ideal for different diseases. Additionally, all biochemical parameters showed significant changes following the treatment. It was shown that high doses of hemp seeds decreased diabetes-induced biochemical damage in rats most probably due to the high content of active compounds. In order to use these seeds in humans, it is essential to find out which hemp compounds are particularly responsible for these effects. Moreover, for the objective investigation of their effects, longer-term studies are needed.

## 1. Introduction

**Figure 1:**
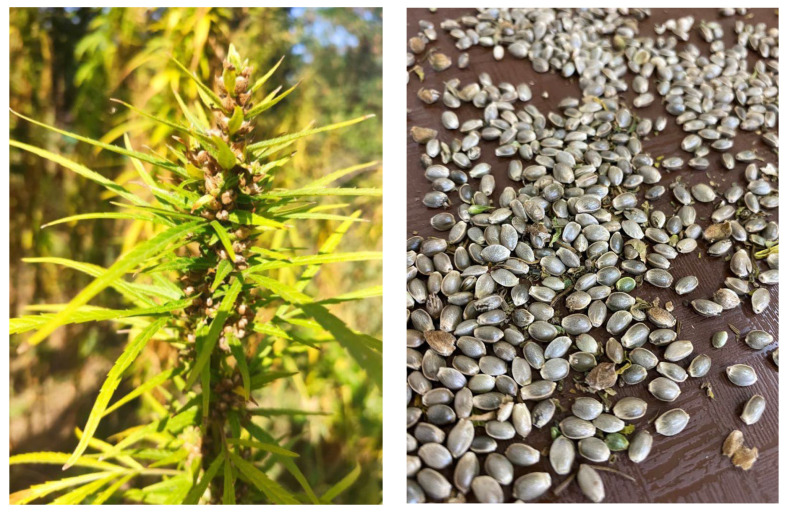
(Original) *Cannabis sativa*, ‘Zenit’ variety.

More than a third of the world’s population is overweight or obese and therefore at risk of developing diabetes mellitus type 2 (T2DM) [1]. This effect has been accentuated since the beginning of the pandemic period, due to the lack of physical activity and the increase in food intake. The disease involves progressive worsening of insulin resistance and a compensatory increase in its secretion. While T2DM is clearly a multifactorial and complex disorder, there is no doubt that obesity-induced insulin resistance accelerates the depletion of pancreatic β cells in the Langerhans Islands and therefore the onset of T2DM [2]. One of the ways to prevent obesity and its associated metabolic disorders is the consumption of plant-based foods with known low caloric values [3]. Moreover, it is of the utmost relevance to note that these products provide a good source of bioactive compounds with beneficial health effects [4]. In this regard, the optimum fiber content, high protein concentration, flavonoid concentration, antioxidant activity, phenol compounds and PUFA omega-6:PUFA omega-3 optimal ratio from industrial hemp seeds illustrate the perfect match [5]. In recent decades, cannabis was banned as it was used for recreational purposes, without a clear distinction regarding the tetrahydrocannabinol (THC) content, which is the psychoactive compound in the plant. The term ‘hemp’ refers to varieties of *Cannabis sativa* that have been grown for industrial purposes and characterized by lower concentrations of THC. According to the Integrated Taxonomic Information System, cannabis belongs to the class Magnoliopsida, order Rosales, family Cannabaceae, genus Cannabis, which has a single species *Cannabis sativa* with the subspecies *sativa* and *indica* [6]. Hemp is a herbaceous and anemophilous plant (Figure 1) native to Central Asia. Due to its long history of cultivation, it is considered one of the oldest cultivated plants. The species was introduced to Europe during the Bronze Age as a cultivated domestic agricultural plant [7]. In the beginning, hemp was cultivated to produce fiber for clothes and seeds for eating, but due to their active compounds, hemp plants are used nowadays in multiple areas besides the earlier ones: in agriculture as a phytoremediation agent of polluted soils, in cosmetics, and as healthy foods, to decrease LDL cholesterol and blood pressure [8,9,10]. From a nutritional perspective, hemp seeds are rich in healthy nutrients such as minerals (zinc, sodium, magnesium, calcium and iron), antioxidants and proteins [11]. Moreover, hemp oil is rich in arginine, alleviating the disrupted metabolism in obesity, regulating blood pressure, and inhibiting the symptoms of type 2 diabetes [12]. In addition, all the essential amino acids can be found in hemp oil [13] alongside high levels of omega-3 fatty acid, α-linolenic acid (ALA), as well as omega-6 fatty linoleic acid (LA). The latter is considered essential because the mammalian body cannot synthesize it and serves as a precursor for the omega-3 group [14]. The ratio between ALA and LA from seeds seems to be perfect for health [15]. This has antioxidant effects on the cardiovascular system overall. Unfortunately, however, hemp oil also contains some trans-fatty acids which are correlated with atherosclerosis due to their role in blocking other PUFA synthesis [16,17]. Additionally, hemp seeds contain gamma-linolenic acid (GLA), with significant antioxidant effects such as the improvement of diabetic complications via anti-inflammatory mechanisms [18]. Other therapeutic antioxidant compounds in hemp seeds are polyphenols [19] and more than 20 flavonoids, of which the most predominant are prenylated flavonoids [20]. Lignanamides and phenol amides have also been observed [21,22]. Their administration is correlated with hypoglycemic effects [23]. Furthermore, in rats with diabetes induced by streptozotocin, hydroxycinnamic acid derivatives, it has been found that hemp seeds significantly decreased blood glucose concentration [24]. Commonly, flavonoids have overall beneficial effects on health due to their involvement in the decrease in the metabolic complications shown in type 2 diabetes [25]. These changes may be the results of amylase inhibition, aiming at the reduction of carbohydrate absorption [25]. Moreover, proteins from the seeds are used for their hypoglycemic effects [26]. For the reasons listed above, the present pilot research investigates the biochemical changes induced by the administration of hemp seed compounds in rats with diabetes. 

## 2. Materials and Methods

### 2.1. Cannabis sativa ‘Zenit’ Variety Hemp Seed Composition

Hemp seeds were collected from plants grown in the Botanical Garden of UASVM Cluj-Napoca. The plants belonged to the ‘Zenit’ variety of *Cannabis sativa* and were used to determine the concentration of lipids, proteins, total phenols, total flavonoid antioxidants activity and quantification of phenols (Figure 1). 

### 2.2. Chemical Analysis

The Kjeldahl method was used for protein content, the Soxhlet method was used for determinations of fat content, while the calcination method served for determining both the dry matter and ash contents [27]. 

Protein content was determined via the Kjeldahl method: mineralization was performed in a Turbotherm TT 265 unit connected to a Turbosorg Tur/K scrubber. A Vapodest 30S unit was used for distillation. All devices were purchased from Gerhardt, Koenigswinter, Germany. Finally, a classic titration was performed, with a 25.00 mL class A burette [27].

Fat content was determined via the Soxhlet method, using a 6-position unit Det Gras N6p (JP Selecta, Barcelona, Spain). Acetone was used as an extraction solvent [27].

The ash content was determined via heating at 550 °C for 5 h in a Nabertherm B180 calcination furnace (Nabertherm GmbH, Lilienthal, Germany) [27].

The dry matter content was determined via heating at 105 °C for 6 h in a forced air oven SLW 53 (POL-EKO-Apparatus, Wodzisław Śląski, Poland).

Total phenols and flavonoids were determined using spectrophotometry VIS/UV/VIS Spectrometer T80+. In this case, the evaluation of the total content of phenols and flavonoids was related to a standard curve of gallic acid. This was dissolved in ethanol. Subsequently, the concentration of total phenols and flavonoids was expressed in mg gallic acid equivalents (GAE) per gram of dried extract [27].

#### 2.2.1. Antioxidant Activity of Hemp Extracts (Methanolic and Ethanolic Extracts)

The antioxidant activity of the samples was determined using the DPPH (1,1-diphenyl-2-picrylhydrazyl) free radical scavenging capacity technique developed by Brand-Williams et al. [28]. To determine sample antioxidant responses, 35 μL of previously methanolic and ethanolic extracted samples mixed with 250 μL of DPPH solution were prepared in triplicate. The reaction solution was incubated for 30 min at room temperature in the darkness before measuring absorbance at 515 nm using a multi-mode plate reader (BioTek, Winuschi, VT, USA). The findings were presented as micromol Trolox equivalents (μmol TE)/100 g sample [28].

#### 2.2.2. Extraction of Phenolic Compounds

To this end, 0.3 g of the crushed sample were extracted with 3 mL of methanol acidified with 1% HCl of 37% concentration, via vortexing for 1 min at the Heidolph Reax top vortex, sonicating for 30 min in the Elmasonic E 15 H sonication bath and centrifuging at 10,000 rpm for 10 min and at 24 °C in the Eppendorf AG 5804 centrifuge. In parallel, the extraction was carried out with 70% ethanol, under the conditions mentioned above. The supernatant was filtered through a 0.45 µm Chromafil Xtra nylon filter and 20 µL were injected into the HPLC system [29].

#### 2.2.3. Fatty Acid Analyses 

Hemp oil extraction was used in this experiment to determine the fatty acids in the seeds. The chemicals used for the preparation of fatty acid methyl esters (FAMEs) were purchased from Sigma-Aldrich (Steinheim, Germany), while the FAMEs standard (37 component FAME Mix, SUPELCO) was from Supelco (Bellefonte, PA, USA). The results obtained were checked against the dry matter. 

Fatty acid methyl esters (FAMEs) of the total lipids (0.2 g oil) were produced via acid-catalyzed transesterification using 1% sulphuric acid in methanol [30,31].

The methylated fatty acids were determined with a gas chromatograph (GC) coupled to a mass spectrometer (MS) (PerkinElmer Clarus 600 T GC-MS; Shelton, CT, USA) [32]. A 0.5 μL sample was injected into a 60 m × 0.25 mm i.d., 0.25 μm film thickness SUPELCOWAX 10 capillary column (Supelco Inc.). The operation conditions were as follows: injector temperature 210 °C; helium carrier gas flow rate 0.8 mL/min; split ratio 1:24; oven temperature 140 °C (hold 2 min) to 220 °C at 7 °C/min (hold 19 min); electron impact ionization voltage 70 eV; trap current 100 μA; ion source temperature 150 °C; mass range 22–395 *m*/*z* (0.14 scans/s with an intermediate time of 0.02 s between the scans). 

The amount of each fatty acid was expressed as area percentages calculated from the total area of identified FAMEs.

### 2.3. Experimental Animal Protocol

Eighteen adult Wistar rats, male and female, weighing between 250 and 300 g were used in the experiment for five weeks. The ethics approval form number was 340/2022. During the experiment, all rats were kept in hygienic conditions: constant temperature and humidity, a light/darkness rhythm of 12/12 h and free access to water and food. The animals were handled gently, without causing them stress or pain. 

In the initial phase, all rats (18 individuals) were kept (with food and water provided ad libitum) until diabetes was induced (2 weeks). They were divided from the beginning into three groups. Both weight and blood glucose were monitored 5 times for two weeks until blood glucose stabilized at values that allowed us to declare induced diabetes. It is important to mention that streptozotocin is a molecule from the nitroso-urea family, used in the treatment of neoplasia or as a natural antibiotic [33]. In 1960, the scientific community found that streptozotocin has selective toxicity on β-cells in the islets of Langerhans of the pancreas. This discovery raised interest in the use of streptozotocin to induce diabetes in experimental animals, especially rats, and mice, but also in rabbits and fish [34,35]. Repeated injections of 20 to 40 mg/kg of streptozotocin in rats and mice decrease insulin secretion without inducing cell death, which mimics insulitis seen in type 1 diabetes. Administered as a single dose of 100 to 200 mg/kg, streptozotocin causes rapid destruction of β-cells and causes diabetes [35]. 

Diabetes mellitus was induced in rats using the Streptozotocin (STZ) protocol previously described [34,35]. Firstly, all the animals were weighted (±1 g) and fasted for 6 h before the start of the experiment. The fasting blood glucose level of each Wistar rat was determined just before the experiment (day 0), using a commercial blood glucose meter (Accu-Chek^®^ instant, Roche, Germany). The rats received a double intraperitoneal injection with STZ (65 mg/kg b.w., dissolved in 0.1 mM citrate buffer, pH 4.5). The rats were returned to their cages and provided with normal food and water. The diabetic state of the rats was evaluated 14 days after the STZ-NA administration by measuring fasting blood glucose levels from blood samples collected from the tail vein. The animals with blood glucose levels of more than 150 mg/dL were considered as diabetic and used in the present study.

Subsequently, they entered the pilot experiment for 5 weeks in which they received normal and specific food. Group one, the control group (CG), individuals without diabetes, and group two, the group with diabetic individuals (DNFG), received water and normal food ad libitum, while the third group, the group with diabetic individuals (DHFG), received specific food-hemp seeds and water ad libitum. 

The experiment was carried out in accordance with the “Guide for the Care and Use of Laboratory Animals” (Department of Health and Welfare Education, National Institute of Health), 1996, and closely followed the directive of the European Council (86/609/1986) and the Order No. 37 of the Romanian Government of 2 February 2002 [36,37]. 

At the end of the experiment, blood samples from all rats were collected for biochemical analyses (0.5 mL). These were performed with microhematocrit tubes. The blood was taken from the orbital sinus into EDTA vacutainers and Eppendorf tubes to obtain plasma and blood serum. According to the literature, in the case of rats, blood glucose values that range between 85 and 132 mg/dL are considered normal [38]. At the beginning of the experiments, all rats had normal glycemia. In the second and third groups, rats received a single dose of the cytotoxic glucose analog intraperitoneally, more precisely STZ (60 mg/kg), freshly dissolved in 10 mM sodium citrate solution (pH = 4.5). The rats with constant blood glucose values of more than 300 mg/dL were considered diabetic [39]. Blood samples were collected from diabetic rats, using the same collection conditions as in all normo-glycaemic rats.

#### Blood Biochemical Parameters

The following parameters were considered: triglycerides (TGs), cholesterol, glucose and fructosamine concentrations using the methods from [40]. The emission spectra were recorded using the Jasco FP-8200 spectrofluorimeter. The method used for the determination of triglycerides was Enzymatic Glycerol Phosphate Oxidase/Peroxidase [41], whereas for determining cholesterolemia, the Enzymatic Cholesterol Oxidase/Peroxidase method was used [42]. As for the blood glucose concentration, the Enzymatic Glucose Oxidase/Peroxidase method was used [43] and for blood fructosamine the Enzymatic glycated-serum-protein assay was used [44].

### 2.4. Statistical Analysis 

GraphPad Prism software was used for statistical analysis. To test the normality data, the Shapiro–Wilk test was used. Parametric (ANOVA) or non-parametric (Kruskal–Wallis) tests were performed to compare the groups. When F was statistically significant (*p* ≤ 0.05), Tukey’s Honest Significant Difference test was applied. Means ± SD were used to express the results.

## 3. Results

### 3.1. The Composition of Cannabis sativa ‘Zenit’, Antioxidant Activity, Identification and Quantification of Phenols and Fatty Acids from Hemp Oil

#### 3.1.1. The Composition of *Cannabis sativa* ‘Zenit’, Antioxidant Activity, Identification and Quantification of Phenols and Fatty Acids from Hemp Oil

Strong causality connections can be found between all the results. Dry matter, lipids, proteins and ash content of hemp seeds are presented in Table 1. The average protein content, 20.95%, and the average lipid content, 29.17%, are the important ones because all the active compounds are proteins and lipids. 

#### 3.1.2. The Composition of Total Phenols and Total Flavonoids 

Analyses of phenols and flavonoids are essential because these substances act as antioxidants, which results in their anti-inflammatory role in various diseases [45]. The results are presented in Table 2 as means ± standard deviation and are expressed in mg GAE/g for phenols (2.75 ± 0.12) and ug CE/g for flavonoids (106.21 ± 5.92).

#### 3.1.3. The Antioxidant Activity

As is seen in Table 3, the antioxidant activity is increased in methanol extract (112.28 μmol Trolox/100 g sample for methanolic extract and 97.09 μmol Trolox/100 g sample for ethanolic extract). Both methods (methanolic and ethanolic extraction) were used to identify the most sensitive for antioxidant identification and quantification. It is shown that the methanolic method is more specific for hemp seeds. 

#### 3.1.4. Phenolic Compounds

As presented in Table 4, 14 phenol compounds were identified and quantified. Lignanamides were found in the largest amount (total lignanamides 2493.17 µg/g hemp seeds for methanol and total 1500.48 µg/g hemp seeds for ethanol) compared to total hydroxycinnamic acid amides (814.78 µg/g hemp seeds for methanol and 444.62 µg/g hemp seeds for ethanol), total hydroxybenzoic acid (504.29 µg/g hemp seeds for methanol and 423.70 µg/g hemp seeds for ethanol) and benzoic acid (93.78 µg/g hemp seeds for methanol and 116.08 µg/g hemp seeds for ethanol). From total lignanamides, we had cannabisins A, B, C, D, F, Q, cannabisin B isomer and grossamide.

#### 3.1.5. Fatty Acid Composition from Seed Oil 

**Figure 2:**
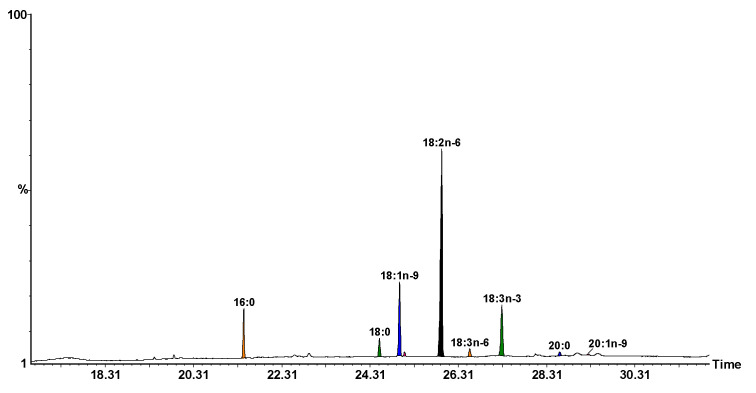
GC-MS chromatogram of FAMEs from hemp seeds analyzed with a Supelcowax 10 capillary column. Peak identification: 16:0, palmitic; 18:0, stearic; 18:1*n*-9, oleic; 18:1*n*-7, vaccenic; 18:2*n*-6, linoleic; 18:3*n*-6, γ-linolenic; 18:3*n*-3, α-linolenic; 20:0, arachidic; 20:1*n*-9, 11-eicosenoic acids.

The results are shown in percentages from areas and presented in Table 5 and Table 6, and Figure 2. As can be observed in Table 6, the highest percentage of fatty acids is represented by polyunsaturated fatty acids, PUFAs, which are crucial, as they are considered indirect antioxidants [46]. The ratio between *n*-6 and *n*-3 (*n*-6/*n*-3), which is the most important for the prevention of various diseases [47] is also presented in Table 6. The *n*-6/*n*-3 ratio is 4.41 and appears to be optimal for preventing and inhibiting disease progression.

### 3.2. Glycemia and Weight during Diabetes Induction in Rats

Glycaemia and weight presented a different trend (Table 7). Both weight and blood glucose were monitored 5 times every two weeks before the stabilization of diabetes. Until the diabetes was stabilized, the blood glucose values varied as seen in Table 7 [48]. Regarding the variation in glucose concentration during diabetes induction, it was shown that the concentrations were higher in both diabetic groups compared to the control group. In contrast, the weight trend was different between means, but the confidence intervals overlap after diabetes induction, namely 246.8 ± 9.2 g in DNFG, and 249.3 ± 17.8 g in DHFG, compared to CG, which had 254.9 ± 17.9.

### 3.3. Blood Biochemical Changes after Hemp Seed Treatment in Diabetic Rats Compared to CG

There were significant differences in glucose and cholesterol concentrations (between the DNFG and DHFG). Regarding triglyceride blood concentration, there were significant changes *p* ≤ 0.05 (*) between the CG and DNFG groups. The cholesterol concentration increased significantly in the diabetic groups compared to the control, with a specification that the hemp seeds decrease the effects of diabetes, *p* ≤ 0.001 (***). As expected, glucose and fructosamine followed the same trend; more significantly, fructosamine had *p* ≤ 0.001, (***) compared to glucose, which had *p* ≤ 0.01 (**), regarding CG and DNFG.

## 4. Discussion

### 4.1. The Composition of Cannabis sativa ‘Zenit’, Antioxidant Activity, Identification and Quantification of Phenols and Fatty Acids from Hemp Oil

As diabetes mellitus represents a major problem for public health, beneficial alternatives are sought in order to prevent the occurrence of this disease [49]. One of them is linked to embracing a healthy lifestyle. In this respect, diet plays a crucial role. The nutritional value of diets is of the utmost importance as daily menus nowadays are poor in nutrients and very rich in calories. In addition, the majority of foods are improved with salts, carbohydrates and fats [50]. A good alternative is to consume more vegetables, fruits, nuts (like hemp seeds), healthy meat, milk and eggs. Therefore, hemp seeds were selected to use in this respect. From the composition of hemp seeds, flavonoids, among others compounds such as lignamides, PUFAs and proteins are recognized for their antidiabetic effects [19]. For instance, flavonoids are involved in the regulation of the insulin-signaling pathway, glucose uptake, as well as adipogenesis [51]. Flavonoids are important antioxidants with antimicrobial, anti-allergic and anti-inflammatory action in both plants and animals. This action is due to their ability to reduce oxidative stress. Moreover, their capacity is doubled by the fact that, once ingested, flavonoids are degraded to phenolic acids, which potentiate this capacity to capture free radicals. 

As such, through their antioxidant role in vivo, they determine the decrease of low-density lipoproteins and restore the homeostasis of polyunsaturated fatty acids in plasma membranes [45]. Moreover, they can have a good influence on β-cell proliferation. In this way, insulin production increases at the same time as hyperglycemia decreases [52]. Phenol compounds such as lignan amides from seeds are capable of anti-inflammatory effects [53]. Since oxidative stress is linked to various pathological conditions, the administration of products with antioxidant activity has been pursued. In this way, as is seen in Table 3, hemp seeds have increased antioxidant capacity [54]. These results may explain health trends from biochemical analyses. Moreover, it is very important to specifically observe which antioxidant compound was responsible for these changes. In this way, the consumption of hydroxybenzoic acid was correlated with hypoglycemic activity [55].

Generally, in plants, lipid composition, and especially fatty acids, differ between taxa. This is due to several factors such as geographical area, soil composition and good environmental conditions [56]. As previously observed, PUFA levels from hemp ‘Zenit’ are higher (Table 6). They are involved in insulin action, membrane cell integrity, cellular-signaling pathway and immune function [57]. According to the literature, there are two groups of long-chain PUFAs: ω-3 and ω-6. The most representative PUFAs are the following: α-linolenic, γ-linolenic, eicosenoic acids, which belong to ω-3, while the ω-6 group includes arachidonic and linoleic acids [58]. As far as PUFAs are concerned, literature data focus on in vitro as well as in vivo studies, which demonstrates that omega-3 acids in particular act as indirect antioxidants in vascular endothelial cells. In this way, they are anti-inflammatory compounds, and therefore their administration reduces the risk of atherosclerosis [46]. As can be observed in Table 5, hemp seeds contain γ-linolenic acid (GLA), which is known for its beneficial effects on health. Firstly, the GLA from the diet is used as a prophylactic compound in different states of some chronic diseases. This is possible due to its ability to change the lipid content from cellular membranes such as prostaglandin E1 and 15-(S)-hydroxy-8,11,13-eicosatrienoic. This change is very healthful because of its antiproliferative and anti-inflammatory effects [59]. Moreover, GLA can be used as treatment for diabetic nephropathy [60]. Regarding the roles of PUFAs, the ratio between two categories of fatty acids needs to be taken into account. For example, in cardiovascular diseases, a 4/1 ratio is correlated with a significant change in mortality, to approximately 70%. At the same time regarding colorectal cancer, a 2.5/1 ratio was associated with a decrease in cell proliferation [47].

### 4.2. Glycemia and Weight during the Experiment

As can be seen in Table 7, there were differences between the weight means of groups, but the confidence intervals overlap (Table 7). This may be due to polyuria determined by diabetes. Additionally, STZ is a toxic substance, and the rats’ appetite might have been lower, especially during diabetes induction. 

A different trend was observed regarding glycemia variation. The increased glucose concentration in DNFG rats compared to CG is due to STZ diabetic effects [61]. As already shown, STZ through alkylation causes the death of β pancreatic cells via glucose transporters 2—GLUT2 [61]. GLUT2s act as sensors and they are responsible for the control of glucose intake by different tissues [62]. Thus, the glucose concentration was different. 

### 4.3. Blood Biochemical Changes after the Treatment with Hemp Seeds in Diabetic Rats

**Figure 3:**
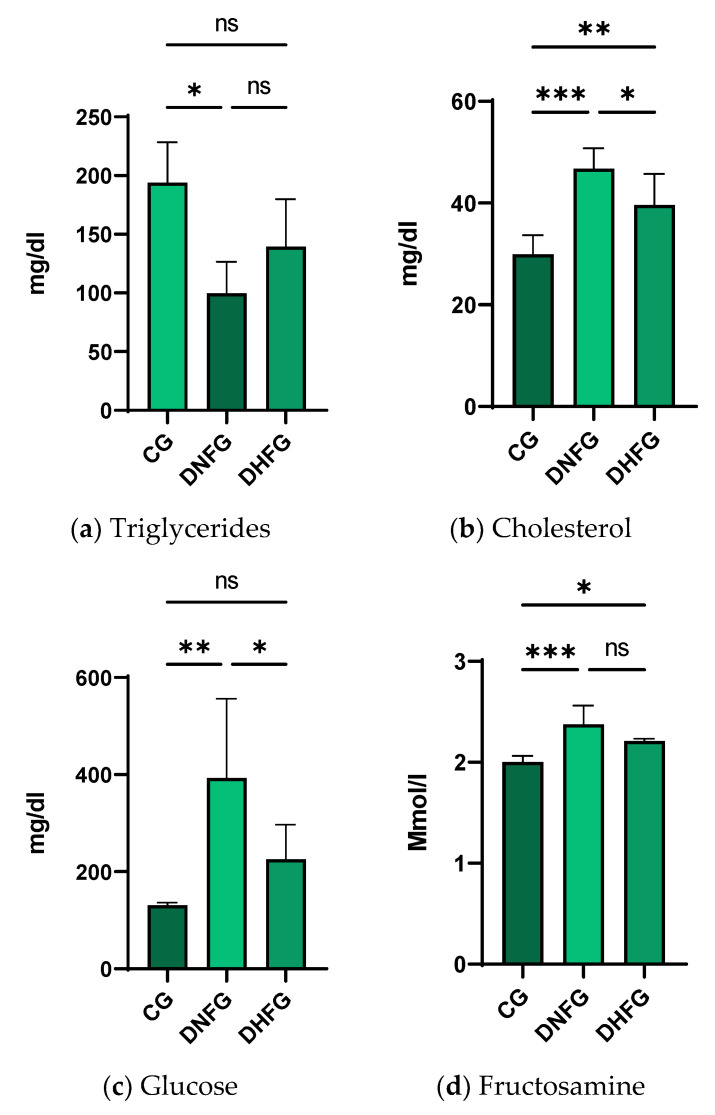
Blood biochemical changes for (**a**) triglyceride, (**b**) cholesterol, (**c**) glucose and (**d**) fructosamine after 5 weeks of treatment in CG, Control Group, which received water and normal feed; DNFG, Diabetic Normal Feed Group—the rats were fed with normal feed; DHFG, Diabetic Hemp Seeds Feed Group—the animals received only hemp seeds as feed. Values are means ± standard deviation (M ± SD), *n* = 6, *p* ≤ 0.05 (*), *p* ≤ 0.01 (**) and *p* ≤ 0.001 (***) (Kruskal–Wallis test for triglycerides and one-way ANOVA for the other parameters).

Biochemical changes are essential because they closely reflect the health of the body. Certain parameters were chosen due to their evolution and usefulness in diabetes assessment (Figure 3). As shown in Figure 3a, a significant decrease in triglyceride concentration was observed after the administration of STZ. On the one hand, STZ administration in rats might have produced a decrease in their appetite and in the case of malnutrition, triglycerides were lowered [63]. This aspect may be supported by the same trend in weight with the same modification observed in children who suffer from protein energy malnutrition. On the other hand, there were studies that reported a high level of triglycerides after diabetes induction of STZ, but following a long period of treatment, from 6 to 12 weeks [64,65]. The rats in this study were treated for five weeks. Hemp seed administration may have increased TG concentration compared to DNFG. The only explanation may be linked to the fact that some compounds from hemp seeds are used as therapeutic agents such as phenolic ones and PUFAs in different diseases and are capable of restoring membrane, structural and biochemical homeostasis [18,66,67]. As expected, cholesterol concentration (Figure 3b) was significantly increased after diabetes induction. Previous studies have shown that this change is normal due to STZ [68]. However, these cholesterol abnormalities can be attributed to insulin deficiency caused by the death of pancreatic β cells. Due to these modifications, cholesterol synthesis is increased. Moreover, the higher rate of LDL glycation is responsible for suppressing cholesterol degradation, which also explains the increase in glycemia after diabetes induction [69]. 

**Figure 4:**
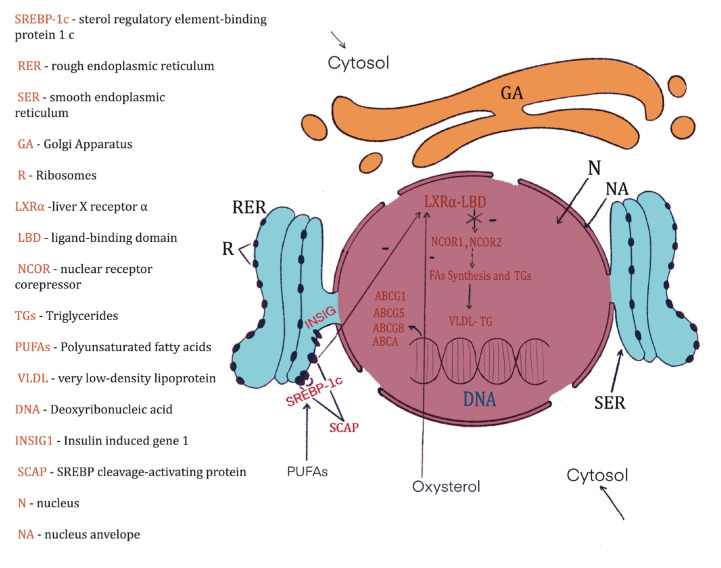
The mechanism by which PUFAs suppress cholesterol synthesis via inhibition of the signaling pathway of sterol regulatory element-binding protein-1 (SREBP-1) [74]. PUFAs through their action on the SCAP complex inhibit LXRα-LBD, which further generates suppression of nuclear corepressor receptors NCOR1 and NCOR2 [75]. All these inhibit the synthesis of fatty acids (FAs) as well as triglycerides (TGs). In this way, VLDL-TG is no longer synthesized. Similar to PUFAs, the high concentration of oxysterol has the same effect; it inhibits LXRα-LBD [76,77].

Considering that the total cholesterol concentration was determined, this variation can be somewhat correlated with the low concentration of triglycerides in the blood. This can be explained by the fact that in the liver, they can bind to VLDL and form VLDL-TG complexes [70]. Interestingly, after the consumption of hemp seeds, cholesterolemia significantly decreased. This significant finding confirms previous studies which showed the same trend in the case of omega-3 fatty acid treatment [71,72]. The mechanism is linked to the capacity of omega-3 fatty acids to stimulate the degradation of apolipoprotein B-100-containing lipoproteins. Finally, cholesterol synthesis is reduced [73]. This is possible due to the mediated capacity of PUFAs, especially omega-3 acids, to suppress the signaling pathway of sterol regulatory element-binding protein-1 (SREBP-1) [74] (Figure 4).

**Figure 5:**
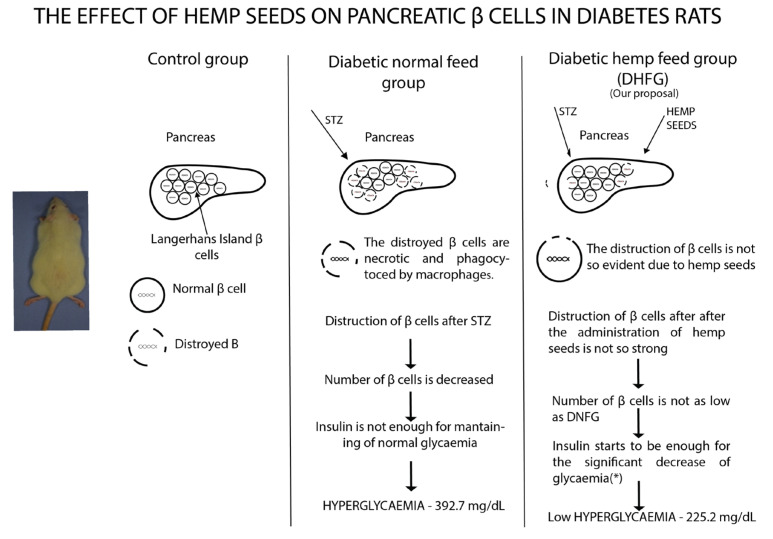
The effect of hemp seeds on pancreatic β cells in diabetes rats. * significant decrease.

As previously reported, STZ administration led to diabetes and in this respect, glycemia was significantly increased in DNFG (Figure 3c) compared to CG [78]. The administration of hemp seeds determined a decrease in glycemia most probably due to their antioxidant content such as phenol compounds, flavonoids and PUFAs, which are recognized for their hypoglycemic effects [18,79] (Figure 5). It is well established that flavonoids are capable of repairing DNA damage. As such, the alkylation induced via STZ may have been suppressed by hydroxycinnamic acid and flavonoid action from hemp seeds [80]. Moreover, Hydroxybenzoic acid has protective effects on pancreatic β cells [81]. In addition, the high level of protein from hemp seeds may be responsible for the decrease in glucose concentration via a mechanism that involves two peptides (Pro-Leu-Met-Leu-Pro, Leu-Arg) which can cause the inhibition of α-glucosidase activity [26]. To some extent, this would still be possible in vivo, only if stem cells were transplanted, and were transformed into pancreatic β cells based on the signaling pathways [82]. As such, glycemia decreased.

The CG, the Control Group, received water and normal food; in the DNFG, the Diabetic Normal Feed Group, the rats were fed with normal feed; in the DHFG, the Diabetic Hemp Seeds Feed Group, the animals received only hemp seeds as feed. Diabetes was induced via streptozotocin (STZ) in Wistar rats. Pancreatic β cells were destroyed. As a result, the number of pancreatic β cells decreased, and the secreted insulin was not sufficient to maintain a normal blood glycemia level. Therefore, all rats had hyperglycemia (392.7 mg/dL). In the rats that ate only hemp seeds, blood glycemia decreased significantly compared to the previous ones (225.2 mg/dL). It is thus assumed that the effects of streptozotocin were inhibited by compounds found in hemp seeds.

Fructosamine (Figure 3d) was used as it is a useful test to verify glucose homeostasis [83,84]. Generally, it is frequently used for the assessment of diabetes mellitus [85]. The term fructosamine is specific for all glycated proteins from serum. The formation of fructosamine compounds is based on a non-enzymatic reaction [86]. As we see in Figure 3d, fructosamine increased after STZ. This change is normal in diabetes and follows the same trend as glycemia [83]. In the DHFG, the consumption of hemp seeds insignificantly decreased fructosamine concentration. Nevertheless, in this case the trend was the same as that of glucose. In this respect, a possible explanation is PUFA hemp seeds. Yoshimura and his coworkers have demonstrated that PUFAs decreased blood glucose concentration as well as fructosamine [87]. Moreover, this modification can be produced via GLA, because it is responsible for insulin sensitivity in rat muscle tissue and ultimately for diabetes suppression [88,89,90].

## 5. Conclusions

In conclusion, our results show that the administration of hemp seeds can modify different biochemical parameters in rats. The significant decreases in glucose and cholesterol concentrations after hemp seed consumption are the most relevant changes in this sense. Once they occur, we can hypothesize that different active compounds, which are found in higher quantity in hemp seeds and are recognized for their anti-diabetic effects, can inhibit pancreatic β-cell destruction, even if the mechanism remains unknown. The high content of antioxidants such as phenol compounds, PUFAs, flavonoids and proteins may be responsible for these health effects. Considering that the present research is a pilot study, it would be necessary to investigate long-term effects with respect to flavonoids, specific phenolic compounds, GLA and PUFAs to find which of them are responsible for these health changes and the mechanisms underlying them. However, hemp seeds may represent a healthy alternative for type 2 diabetes. Extrapolating the results to other animals and humans will represent a necessary requirement.

## Figures and Tables

**Table 1:** *Cannabis sativa* ‘Zenit’ composition.

Analyses	Dry Matter (%)	Lipid Content (%)	Protein Content (%)	Ash Content (%)
Mean ± SD	94.57 ± 0.16	29.17 ± 0.46	20.95 ± 0.21	4.61 ± 0.33

The results obtained were checked against the dry substance; all the results are expressed as means ± standard deviation (M ± SD); SD = standard deviation.

**Table 2:** The composition of total phenols and total flavonoids.

Type of Analyses	Total Phenols	Total Flavonoids
UM	2.75 ± 0.12 mg GAE/g	106.21 ± 5.92 μg CE/g

UM—unit of measurement; the results are presented as means ± standard deviation (M ± SD). GAE, milligrams of gallic acid equivalents per gram; CE, micrograms catechin equivalents per gram.

**Table 3:** Antioxidant activity of hemp extracts (methanolic and ethanolic extracts).

Samples	uM Trolox/100 g (Mean ± SD)
Methanol extract	112.28 ± 5.61 μmol Trolox/100 g sample
Ethanolic extract	97.09 ± 4.85 μmol Trolox/100 g sample

The values are expressed as micromol Trolox equivalents (μmol TE)/100 g sample. The results are presented as means ± standard deviation (M ± SD).

**Table 4:** Identification and quantification of phenolic compounds in the hemp seed sample (µg/g hemp seeds) via HPLC-DAD-MS-ESI+ analysis.

Peak	R_t_ (min)	UV λ_max_ (nm)	[M + H]^+^ (m/z)	Phenolic Compound	Subclass	Hemp Seeds Methanol (Mean ± SD)	Hemp Seeds Ethanol (Mean ± SD)
1	2.78	270	123	Benzoic acid	Benzoic acid	93.78 ± 9.40	116.08 ± 11.20
2	2.97	270	139	2-Hydroxybenzoic acid	Hydroxybenzoic acid	210.06 ± 10.20	271.33 ± 13.57
3	9.47	280	155	Protocatechuic acid	Hydroxybenzoic acid	294.23 ± 14.56	152.37 ± 7.60
4	10.87	290	595	Cannabisin A	Lignanamide	82.68 ± 7.34	56.11 ± 4.80
5	11.48	290	597	Cannabisin B	Lignanamide	130.05 ± 10.07	149.54 ± 12.46
6	11.78	320,290	284	*N-trans*-Coumaroyltyramine	Hydroxycinnamic acid amide	196.91 ± 9.85	172.56 ± 8.63
7	14.01	290	611	Cannabisin C	Lignanamide	179.65 ± 8.98	203.55 ± 10.15
8	14.62	290	597	Cannabisin B isomer	Lignanamide	153.96 ± 7.50	146.88 ± 7.23
9	18.20	322,290	301	*N-trans*-Caffeoyltyramine	Hydroxycinnamic acid amide	339.04 ± 16.95	181.42 ± 9.07
10	19.29	340,290	597	Cannabisin Q	Lignanamide	201.34 ± 10.03	171.67 ± 8.85
11	20.40	330,290	314	*N*-Feruloyltyramine	Hydroxycinnamic acid amide	278.83 ± 11.15	90.65 ± 8.53
12	22.09	290	625	Cannabisin D	Lignanamide	444.42 ± 17.71	119.87 ± 10.20
13	22.79	290	625	Cannabisin F	Lignanamide	470.99 ± 23.50	231.01 ± 11.55
14	23.39	320,290	625	Grossamide	Lignanamide	830.08 ± 41.50	421.84 ± 21.09
				Total phenolics		3906.02	2484.88

**Table 5:** The specific fatty acids from *Cannabis sativa* seed oil.

Specific Fatty Acids	Concentration (% by Area)	Standard Deviation (SD)
16:0	7.84	0.27
18:0	3.68	0.15
18:1*n*-9	15.73	0.66
18:1*n*-7	0.71	0.05
18:2*n*-6	55.93	2.49
18:3*n*-6	1.73	0.08
18:3*n*-3	13.06	0.58
20:0	0.97	0.06
20:1*n*-9	0.35	0.03

16:0, palmitic; 18:0, stearic; 18:1*n*-9, oleic; 18:1*n*-7, vaccenic; 18:2*n*-6, linoleic; 18:3*n*-6, γ-linolenic; 18:3*n*-3, α-linolenic; 20:0, arachidic; 20:1*n*-9, 11-eicosenoic acids. The results are presented in percentages. The values represent the means of three samples, analyzed individually in triplicate (*n* = 3 × 3). SD—standard deviation.

**Table 6:** Fatty acid type from *Cannabis sativa* seed oil.

Fatty Acid Type	Concentration (% by Area)	Standard Deviation (SD)
SFA	12.49	0.61
MUFA	16.78	0.69
PUFA	70.72	2.97
*n*-3 PUFA	13.06	0.59
*n*-6 PUFA	57.66	2.57
*n*-6/*n*-3	4.41	
PUFAs/SFAs	5.66	

SFAs, saturated fatty acids; MUFAs, monounsaturated fatty acids; PUFAs, polyunsaturated fatty acids. The values represent the means of three samples, analyzed individually in triplicate (*n* = 3 × 3). SD—standard deviation.

**Table 7:** Glucose and weight prior to the experiment.

Parameters	CG	DNFG	DHFG
Glycemia (mg/dL)	112 ± 11.9	422.3 ± 120.2	398.8 ± 98.9
Weight (g)	254.9 ± 17.9	246.8 ± 9.2	249.3 ± 17.8

CG, the Control Group, received water and normal food; in the DNFG, the Diabetic Normal Feed Group, the rats were fed with normal feed; in the DHFG, the Diabetic Hemp Seeds Feed Group, the animals received only hemp seeds as feed; the values are presented as mean ± standard deviation (M ± SD), *n* = 6.

## Data Availability

Data is contained within the article.
